# SCALP MENINGIOMA

**DOI:** 10.4103/0019-5154.44799

**Published:** 2008

**Authors:** Sunil K Singh, B K Ojha, A Chandra, M Rastogi, M Husain, N Husain

**Affiliations:** *From the Department of Neurosurgery, King George's Medical University, Lucknow - 226 003, India*; 1 *From the Department of Neuropathology, King George's Medical University, Lucknow - 226 003, India*

**Keywords:** *Ectopic*, *meningioma*, *scalp*, *transitional*

## Abstract

Primary extracranial meningiomas occur very rarely. We present a rare case of extracranial meningioma of the transitional variant which was excised satisfactorily. There was no suggestion of any connection to the intracranial compartment or cranial nerves. The underlying galea was uninvolved, suggesting the true extracranial nature of this tumour. This rare diagnosis should nonetheless be kept in the differential diagnosis of scalp tumors.

## Introduction

Extracranial meningiomas are extremely rare tumours and constitute less than 2% of all meningiomas. Common sites of reported occurrence are skin, orbit, paranasal sinuses, temporal fossa and oral cavity. Other sites include nasal cavity, parotid gland, bifurcation of carotid artery, little finger of right hand, brachial plexus, foot, scalp, face and paravertebral region.^1^,^2^ They have been variously referred to as ectopic, extradural, calvarial, cutaneous, extraneuraxial or intraosseous meningiomas. To avoid this confusion, Lang *et al*. have proposed the term “primary extradural meningioma” (PEM) for such lesions.

Various classification schemes have been proposed to classify these tumors^3^,^5^–^7^,^9^,^12^ (Table 1).

**Table 1:** Classification schemes for extra cranial meningiomas

Hoye classification	Lopez classification	Lang classification
Primary: extracranial meningioma arising in a neural foramina	Primary: occur in children and young adults and are usually present since birth	Lesions that were purely extracalvarial with no attachment to bone
Secondary: extracranial extension of an intracranial meningioma	Represent a cutaneous extension from an ectopic soft tissue meningioma	Purely calvarial, being located entirely within the bone of the skull (B = skull base; C = convexity)
Ectopic: without any connection to a foramina or intracranial structure	Represent an extension into the skin from CNS meningioma infiltrating across bone or a bone defect	Calvarial tumors with extracalvarial extension (B = skull base; C = convexity)
Metastatic: extracranial metastasis of an intracranial meningioma

## Case Report

An 18-year-old patient was referred to us with history of a slowly growing small nodule on the right posterior frontal area of scalp since infancy (present size 5 cm × 7 cm × 4 cm). Massive bleeding had aborted a previous attempt at excision one month back. This was followed by ulceration and foul discharge.

On examination, a large fleshy ulcerated mass was present over the right posterior frontal region, which was firm, nontender and freely mobile over the periosteum. The overlying skin could not be pinched up separately. The surrounding skin was normal, with no associated lymphadenopathy.

General physical examination was normal. Contrast-enhanced CT scan of the head showed an extracranial soft tissue mass with calcification over the right posterior frontal region without any evidence of involvement of the calvarium or intracranial structures.

The mass was excised with 2.5-cm margins, and the defect was closed with split thickness skin graft. The mass had well-defined margins and the tumour did not involve the periosteum.

On histopathological examination, the deeper dermis and subcutaneous fat were infiltrated by a tumor composed of syncytial as well as whorls of spindle cells with poorly defined cell borders around blood vessels. The nuclei were elongated to oval with finely distributed chromatin and inconspicuous nucleoli. Some nuclei appeared vesicular due to cytoplasmic inclusions. Abundant psammoma body formations were also seen with areas of collagenization. The findings were suggestive of *transitional meningioma*.

## Discussion

Most of the reported primary extradural meningiomas have been of meningothelial or psammomatous origin,^1^ although some authors have reported the fibroblastic variety to be more common.^11^ It is interesting to note that extensive search of accessible literature revealed that ectopic transitional meningioma has not yet been described. It is generally agreed that meningiomas originate from meningiocytes (arachnoid cells or meningothelial cells) capping the arachnoid villi. However, clusters of arachnoidal cells have been found in the sheaths of the cranial and spinal nerves at their exit from the skull and vertebrae. The presence of such cells has also been suggested in the cranial periosteum. It is also theoretically possible that some ectopic meningiomas may be derived from perineurial cells rather than from displaced arachnoid cells. Heterotopic brain and meningeal tissue is known to occur occasionally in the midline of head, neck and trunk due to displacement of such tissue during the fusion of skull and spine in the embryonic state, which may be a source for development of ectopic meningiomas.^1^,^8^,^9^

The lesion may be mistaken clinically for cutaneous lesions including cysts, skin tag, nevi, vascular lesions, and fibroma. It might be associated with circumscribed alopecia, congenital melanocytic nevus, adenomatous hyperplasia of the eccrine glands and with congenital localized hypertrichosis (hair tufts). Association with von Recklinghausen's disease and malformations of fingers and toes and ovarian fibroma have also been reported.^13^

Most of the ectopic meningiomas had occurred within the orbit, probably originating from the arachnoid cells in the sheath of the optic nerve.^4^ In the present case, the diagnosis of primary ectopic meningioma was based on the fact that there was no clinical and radiological evidence of an intracranial lesion. In an earlier study, the initial diagnosis was made on the basis of FNAC, while the final HPE confirmed the diagnosis.^10^ In our study, the final excisional biopsy report was of transitional meningioma.

**Fig. 1:**
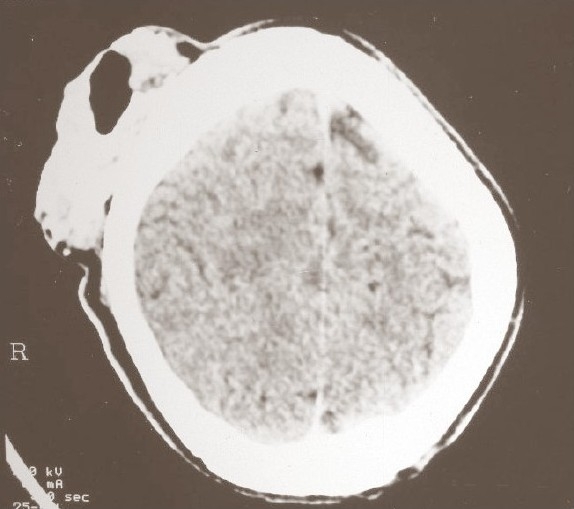
Pre-operative CECT showing an extracalvarial soft tissue mass without any underlying pathology

**Fig. 2:**
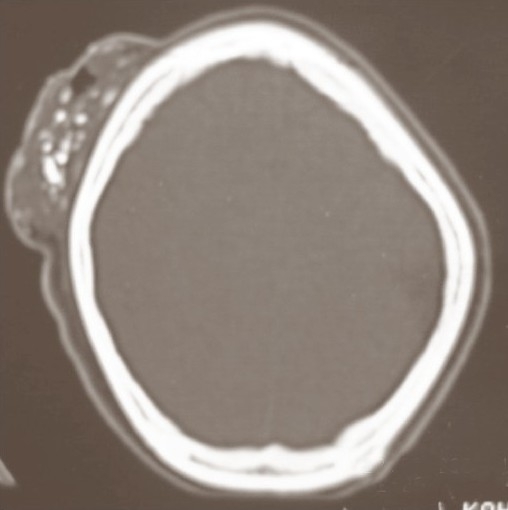
Bone window at the same level as that in Fig. 1, showing stippled calcification within the lesion. The underlying bone is normal

**Fig. 3:**
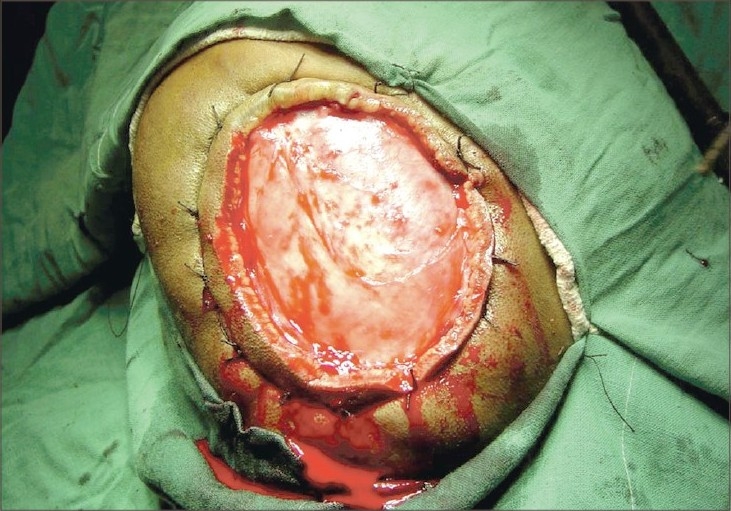
Operative view after excision

**Fig. 4:**
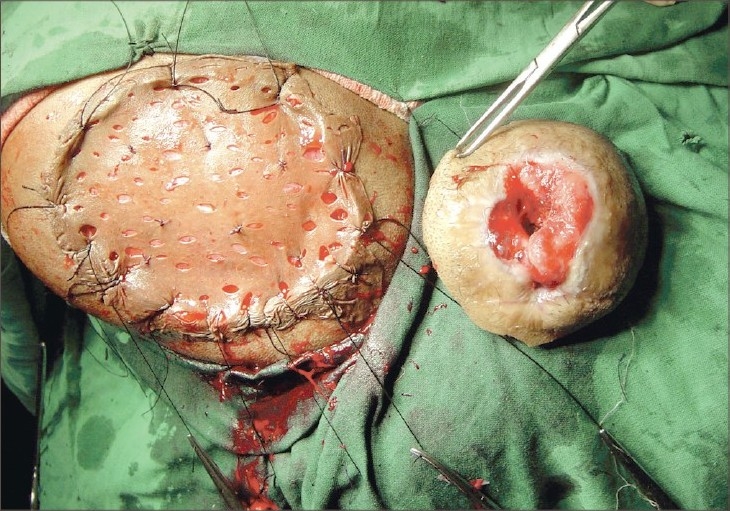
Per-operative view after excision of the lesion and skin grafting

**Fig. 5:**
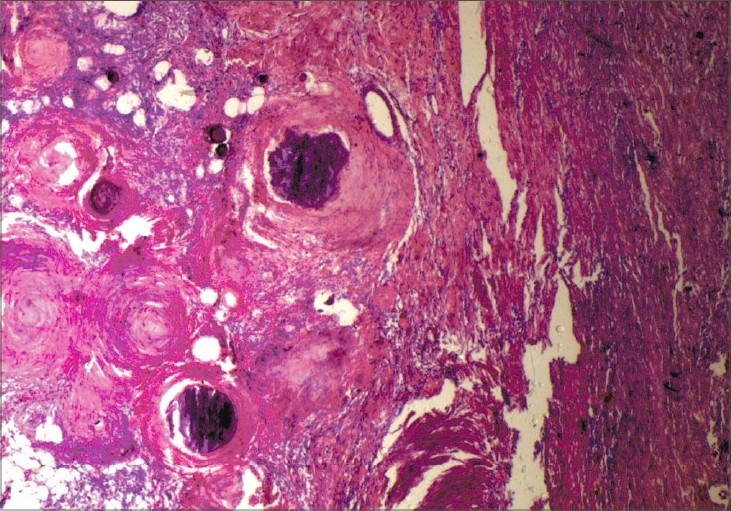
Histopathological slide (H & E ×400 stain) – tumor cells within the dermis with interspersed Verrocay bodies, suggestive of a transitional meningioma

**Fig. 6:**
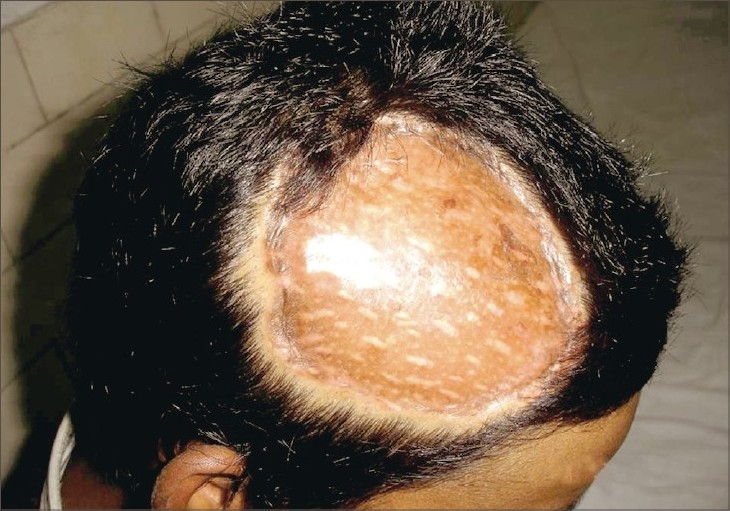
Photograph at 6 months follow up

## References

[1] Marthandapillai A, Alappat JP (2000). Ectopic meningioma: A case report. Neurol India.

[2] Tomaru U, Hasegawa T, Hasegawa F, Kito M, Hirose T, Shimoda T (2000). Primary extra cranial meningioma of the foot: A case report. Jpn J Clin Oncol.

[3] Munjal M, Passey JC, Sethi A, Singhal D, Bansal R (2005). Primary extra cranial meningioma as a part of neurofibromatosis-2 in head and neck: A rare case report. Int J Otorhinolaryngol.

[4] Goel A, Mehta A, Gupta S (1999). Unusual mode of spread and presentation of meningioma: A case report. Neurol India.

[5] Hoye SJ, Hoar CS, Murray JE (1960). Extacranial meningioma presenting as a tumor of the neck. Am J Surg.

[6] Thamburaj VA Intracranial meningiomas. Neurosurgery on the web.

[7] Lang FF, Macdonald OK, Fuller GN, DeMonte F (2000). Primary extradural meningiomas: A report on nine cases and review of literature from the era of computerized tomography scanning. J Neurosurg.

[8] Azar-Kia B, Sarwar M, Marc JA, Schechter MM (1974). Intraosseous meningioma. Neuroradiology.

[9] Lopez DA, Silvers DN, Helwig EB (1974). Cutaneous meningiomas: A clinicopathologic study. Cancer.

[10] Kalfa M, Daskalopoulou D, Markidou S (1999). Fine needle aspiration (FNA) biopsy of primary cutaneous meningioma: Report of two cases. Cytopathology.

[11] Miyamoto T, Mihara M, Hagari Y, Shimao S (1995). Primary cutaneous: Meningioma on the scalp: Report of two siblings. J Dermatol.

[12] Sharma JK, Pippal SK, Sethi Y (2006). A rare case of primary Nasoethmoidal meningioma. Indian J Otolaryngol Head Neck Surg.

[13] Simic R, Duricic S, Plamenac P, Ilic S, Todarovic V (2001). Cutaneous meningioma of the scalp in an infant. Arch Oncol.

[14] Sibley DA, Cooper PH (1989). Rudimentary meningocele: A variant of “primary cutaneous meningioma. J Cutan Pathol.

